# Supplementary material for: SARS-CoV-2 ORF3a Induces Incomplete Autophagy via the Unfolded Protein Response
Source: Viruses. 2021 Dec 9;13(12):2467. doi: 10.3390/v13122467 (PMC8706696; doi:10.3390/v13122467)

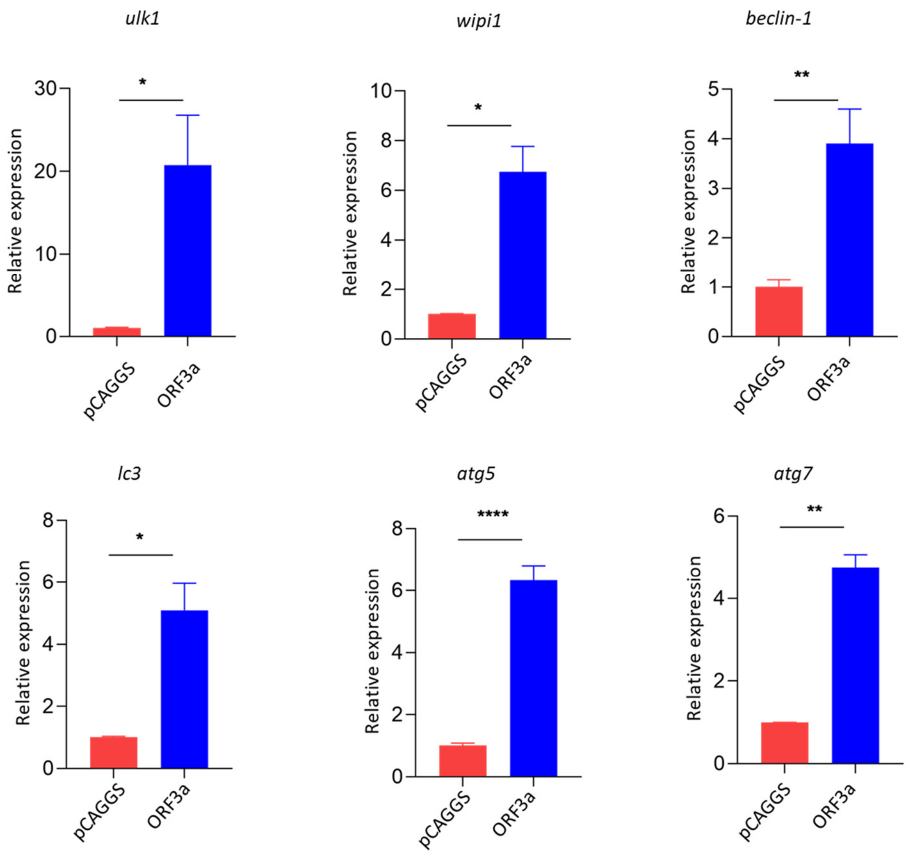

Figure. S1. SARS-CoV-2 ORF3a promoted the expression of autophagy-related genes.

Vero-E6 cells (1-2*10^5 cells) were transfected with HA-Tag-ORF3a (2 µg) or empty vector (2 µg) for 24 h. qPCR was performed to detect the expression level of *ulk1*, *wipi1*, *beclin-1*, *Atg5*, *Atg7*, and *lc3*.

Data were analyzed by T-tests or T-tests with Welch's correction. qPCR Data (mean ± SEM) were representative of three independent experiments. (*$p \le 0.05$, **$p \le 0.01$, ****$p \le 0.001$)

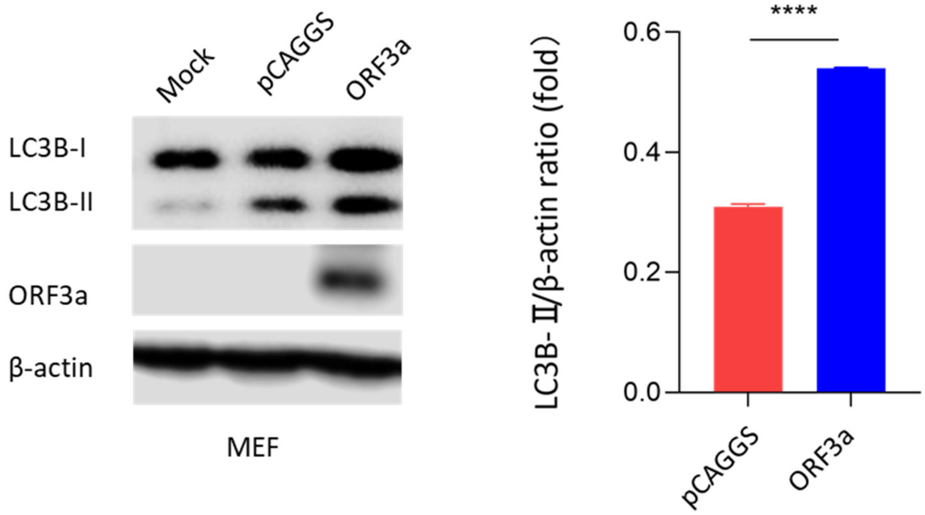

Figure. S2. ORF3a could induce autophagy.

MEF cells (1-2*10^5 cells) were transfected with HA-Tag-ORF3a (2 μg) or empty vector (2 μg) for 24 h. Western blot was performed to detect LC3B.

Data were analyzed by T-tests. Data were representative of three independent experiments. (, ****$p \leq 0.001$)

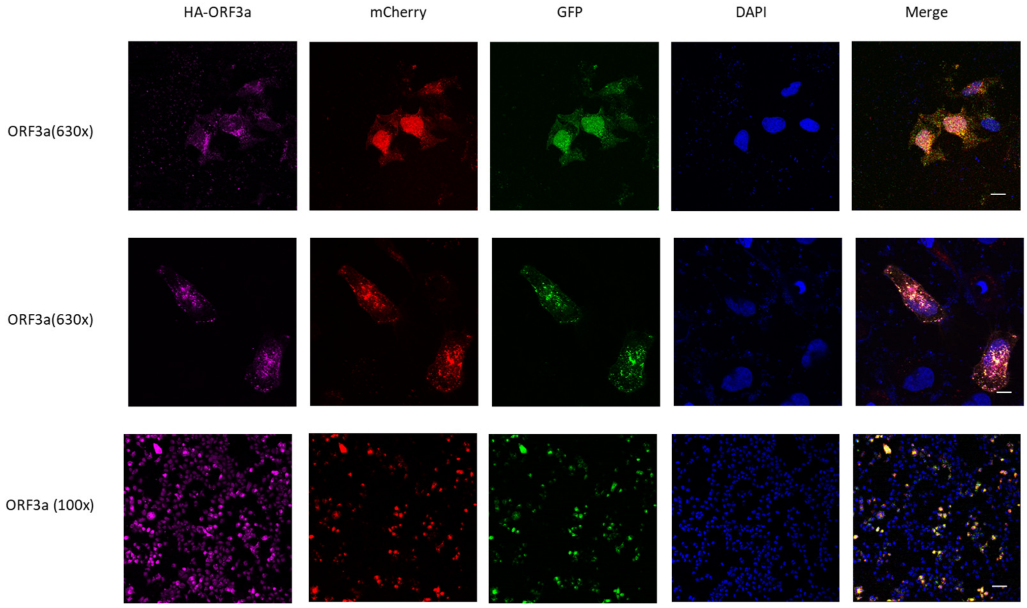

Figure. S3. ORF3a-induced autophagy was an incomplete process.

Hela cells (1-2*10^5 cells) were co-transfected with mCherry-GFP-LC3B (1 μg) and

HA-Tag-ORF3a (1 μg) for 24 h. Immunofluorescence was performed to assess autophagy status. Scale bars: 4μm.

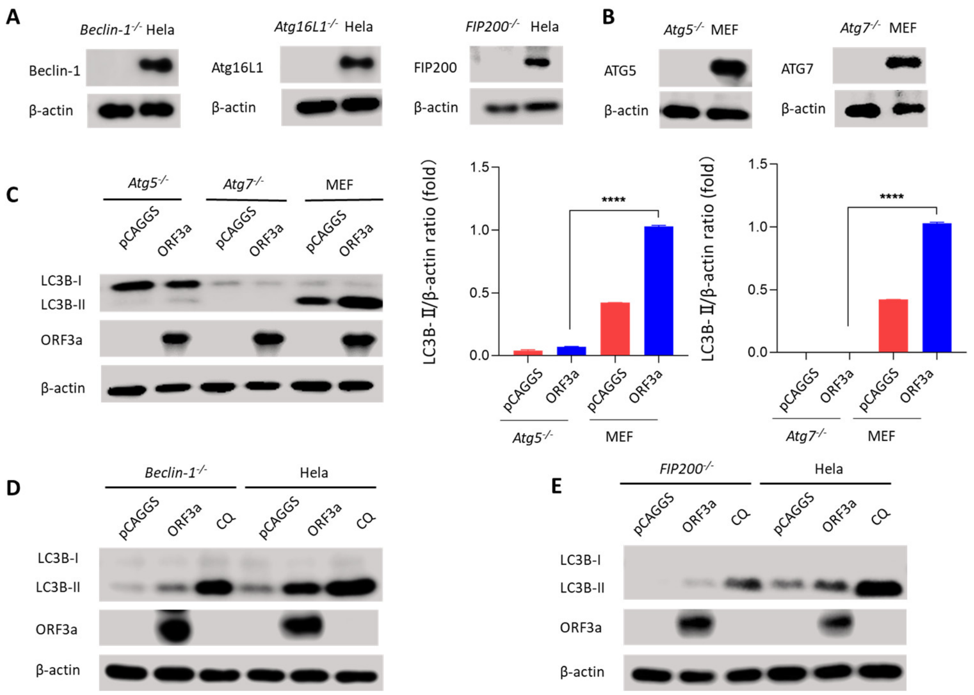

Figure. S4. ORF3a-induced autophagy was classic.

(A) *Beclin-1*, *Atg16L1*, and *FIP200* KO Hela cells (1-2*10^5 cells) were lysed to verify successful knockout.

(B) *Atg5* and *Atg7* KO MEF cells (1-2*10^5 cells) were lysed to verify successful knockout.

(C) *Atg5* KO, *Atg7* KO, and WT MEF cells (1-2*10^5 cells) were transfected with HA-Tag-ORF3a (2 μg) or empty vector (2 μg) for 24 h. Western blot was performed to detect LC3B.

(D and E) Hela cells (1-2*10^5 cells) and *Beclin-1* / *FIP200* KO Hela cells (1-2*10^5 cells) were transfected with HA-Tag-ORF3a (2 μg) or empty vector (2 μg) for 24 h or treated with CQ (50 nM) for 6 h. Western blot was performed to detect LC3B.

One-way ANOVAs followed by Dunnett's multiple comparisons test were used in comparing multiple groups. Data were representative of 3 independent experiments. (*****$p \leq 0.001$*)

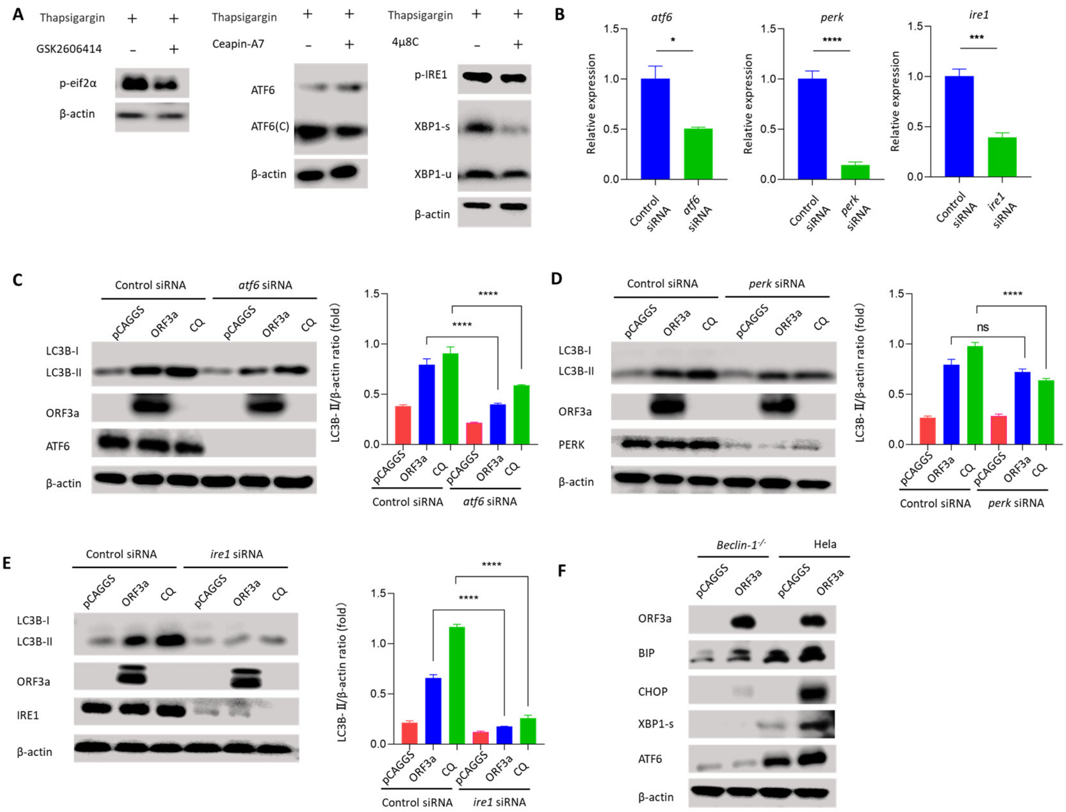

Figure. S5. ORF3a induced autophagy through unfolded protein response.

(A) 293T cells (1-2*10^5 cells) were pretreated with Ceapin-A7 (10 nM), GSK2606414 (5 nM), 4μ8C (10 nM) for 24 h and treated with Thapsigargin (2 μM) for 2 h before harvest. Western blot was performed to detect relative UPR proteins.

(B) Hela cells (1-2*10^5 cells) were transfected with atf6 / perk / ire1 siRNA (50 nM) or control siRNA (50 nM) for 24 h. qPCR was performed to detect the expression level of *atf6*, *perk*, *ire1*.

(C, D and E) Hela cells (1-2*10^5 cells) were transfected with HA-Tag-ORF3a (2 μg) or empty vector (2 μg) and atf6 / perk / ire1 siRNA (50 nM) or control siRNA (50 nM) for 36 h. Hela cells (1-2*10^5 cells) were transfected with atf6 / perk / ire1 siRNA (50 nM) or control siRNA (50 nM) for 36 h and treated with CQ (50 nM) for 6 h before harvest. Western blot was performed to detect LC3B.

(F) Hela cells (1-2*10^5 cells) and *Beclin-1* KO Hela cells (1-2*10^5 cells) were transfected with HA-Tag-ORF3a (2 μg) or empty vector (2 μg) for 24 h Western blot was performed to detect relative UPR proteins.

Data were analyzed by T-tests or T-tests with Welch's correction. One-way ANOVAs followed by Dunnett's multiple comparisons test were used in comparing multiple groups. Data were representative of three independent experiments. ($*p \leq 0.05$, $***p \leq$

0.001, ****$p \leq 0.001$)

Supplementary Table S1.

Gene ID	Gene Symbol	Species	Sequence 5' to 3'
60	*β-actin*-F	human	CATGTACGTTGCTATCCAGGC
60	*β-actin*-R	human	CTCCTTAATGTCACGCACGAT
8408	*ulk1*-F	human	GGCAAGTTCGAGTTCTCCCG
8408	*ulk1*-R	human	CGACCTCCAAATCGTGCTTCT
9474	*atg5*-F	human	AAAGATGTGCTTCGAGATGTGT
9474	*atg5*-R	human	CACTTTGTCAGTTACCAACGTCA
10533	*atg7*-F	human	CAGTTTGCCCCTTTTAGTAGTGC
10533	*atg*7-R	human	CCAGCCGATACTCGTTCAGC
8678	*beclin-1*-F	human	CCATGCAGGTGAGCTTCGT
8678	*beclin-1*-R	human	GAATCTGCGAGAGACACCATC
81631	*lc3*-F	human	GATGTCCGACTTATTCGAGAGC
81631	*lc3*-R	human	TTGAGCTGTAAGCGCCTTCTA
9776	*atg13*-F	human	TTGCTATAACTAGGGTGACACCA
9776	*atg13*-R	human	CCCAACACGAACTGTCTGGA
55062	*wipi1*-F	human	ACTAAAGCCGGGTATAAGCTGT
55062	*wipi1*-R	human	CGGGATTTCATTGCTTCCGTG
22241	*ulk1*-F	mouse	AAGTTCGAGTTCTCTCGCAAG
22241	*ulk1*-R	mouse	ACCTCCAGGTCGTGCTTCT
56208	*beclin-1*-F	mouse	ATGGAGGGGTCTAAGGCGTC
56208	*beclin-1*-R	mouse	TGGGCTGTGGTAAGTAATGGA
55062	*wipi1*-F	mouse	CTGCTTCTCTTTCAACCAAGACT
55062	*wipi1*-R	mouse	ACGTCAGGGATTTCATTGCTT
67443	*lc3*-F	mouse	TTATAGAGCGATACAAGGGGGAG
67443	*lc3*-R	mouse	CGCCGTCTGATTATCTTGATGAG
74244	*atg7*-F	mouse	TCTGGGAAGCCATAAAGTCAGG
74244	*atg7*-R	mouse	GCGAAGGTCAGGAGCAGAA
11793	*atg5*-F	mouse	TGTGCTTCGAGATGTGTGGTT
11793	*atg5*-R	mouse	ACCAACGTCAAATAGCTGACTC
11461	*β-actin*-F	mouse	GGCTGTATTCCCCTCCATCG
11461	*β-actin*-R	mouse	CCAGTTGGTAACAATGCCATGT
22926	*atf6*-F	human	TCCTCGGTCAGTGGACTCTTA
22926	*atf6*-R	human	CTTGGGCTGAATTGAAGGTTTTG
9452	*perk*-F	human	ACGATGAGACAGAGTTGCGAC
9452	*perk*-R	human	ATCCAAGGCAGCAATTCTCCC
2081	*ire1*-F	human	CACAGTGACGCTTCCTGAAAC
2081	*ire1*-R	human	GCCATCATTAGGATCTGGGAGA

Supplementary Table S2.

Gene ID	Gene Symbol	Species	Sequence 5' to 3'
9452	*siperk*-1-F	human	GCCAUAAUGGACAUAGUGATT
9452	*siperk*-1-R	human	UCACUAUGUCCAUUAUGGCTT
9452	*siperk*-2-F	human	CAAUCUUGCAGUAUCCAUATT
9452	*siperk*-2-R	human	UAUGGAUACUGCAAGAUUGTT

9452	*siperk*-3-F	human	CCUCAAGCCAUCCAACAUAUU
9452	*siperk*-3-R	human	AAUAUGUUGGAUGGCUUGAGG
2081	*siire1*-1-F	human	CCCAUCAACCUCUCUUCUGUA
2081	*siire1*-1-R	human	UACAGAAGAGAGGUUGAUGGG
2081	*siire1*-2-F	human	CAUCGUUCACAGAGACCUATT
2081	*siire1*-2-R	human	UAGGUCUCUGUGAACGAUGTT
2081	*siire1*-3-F	human	CGAUCGUGAAGCAGUUAGATT
2081	*siire*1-3-R	human	UCUAACUGCUUCACGAUCGTT
22926	*siatf6*-1-F	human	GCAGCAACCAAUUAUCAGUUU
22926	*siatf6*-1-R	human	AAACUGAUAAUUGGUUGCUGC
22926	*siatf6*-2-F	human	GCUGGAUGAAGUUGUGUCATT
22926	*siatf6*-2-R	human	UGACACAACUUCAUCCAGCTT
22926	*siatf6*-3-F	human	GGUUCAUAGACAUGAAGUATT
22926	*siatf6*-3-R	human	UACUUCAUGUCUAUGAACCTT

Supplementary clone map.

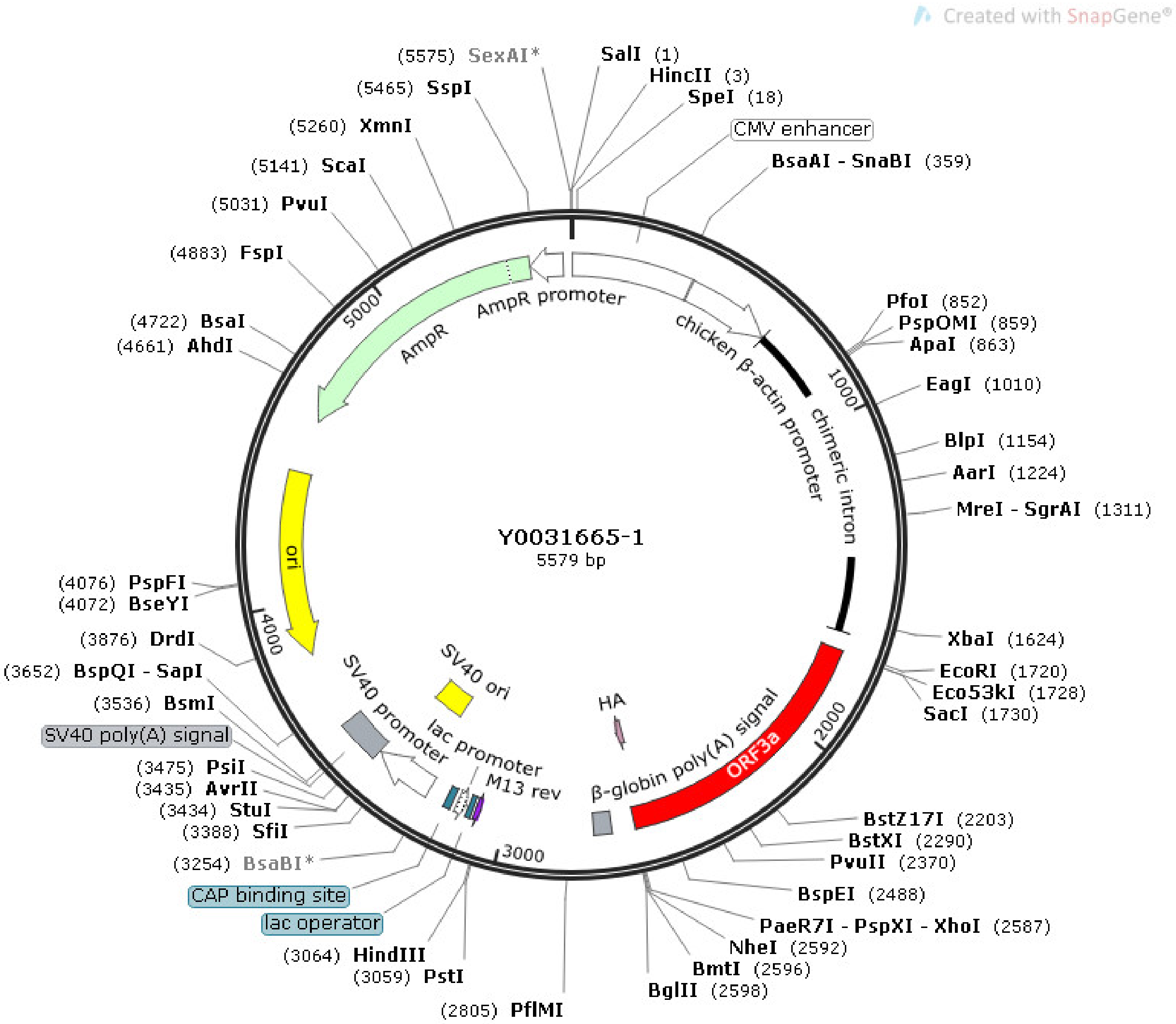

Supplement: Supplementary file 1 [file viruses-13-02467-s001.zip › viruses-1476722-supplementary.pdf]
